# Improving access to refractive and eye health services

**Published:** 2015

**Authors:** Luigi Bilotto, Michael Morton, Luisa Casas Luque, Rob Terry, Judith Stern, Kesi Naidoo

Avoidable blindness and vision impairment affects 741 million people worldwide. Uncorrected refractive error (LIRE) is the major cause, accounting for 84% of all cases, or 625 million people.^1^ Despite LIRE being easily detected, measured and corrected, many countries have inadequate refractive services^2^, in large part due to the limited number of relevant eye care personnel and a lack of health systems integration.^3^

## Human capital

The World Health Organization (WHO) Universal Eye Health Global Action Plan 2014–2019^4^ recognises that more human resources in optometry are required to reach the targets that have been set. Globally, there is an eye health workforce equating to about 167,000 clinical refractionists (e.g. optometrists, ophthalmologists, opticians, refractionists, ophthalmic nurses) providing refractive services full-time. Based on the WHO'S conservative requirements of 1 functional clinical refractionist per 50,000 persons^5^, an additional 47,000 eye care workers (providing refractive services full time) would be required to fulfil the VISION 2020 human resource target. However, taking into account the global prevalence of refractive error (>50%), considering a more suitable population coverage of 1 per 10,000, and correcting for some assumptions used to calculate that figure, we estimate that the world would need over 1 million functional clinical refractionists to address the LIRE challenge. Optometrists are a key professional group involved in the management of LIRE, particularly in low- and middle-income countries. The training of optometrists is therefore critical to creating the required human capacity in refractive care.

## Education as a solution

The WHO has called for the development and maintenance of a sustainable eye care service workforce as well as training and career development for eye health professionals.^4^ The development of a refractive error workforce therefore requires both of the following:

- **Optometry development.** Optometry schools and services must be developed (or reinforced) within existing systems
- **Eye health education.** Existing eye health workers within the health care system must be up-skilled (in specific skills or competency levels) in order to promote the delivery of quality comprehensive services.

**Figure:**
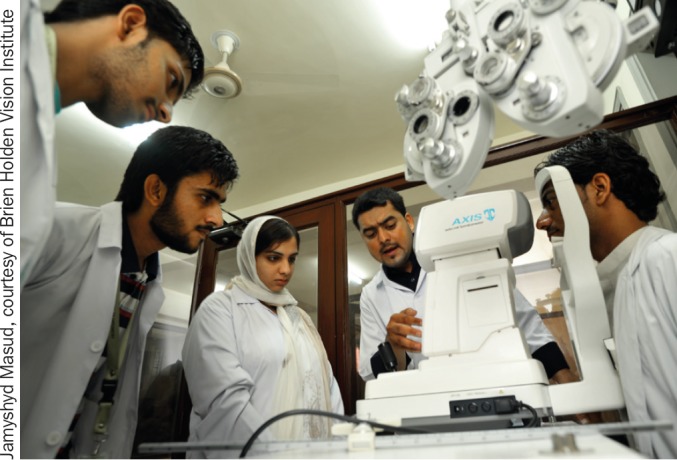
Refraction training. PAKISTAN

## Optometry development

The sustainable development of optometric education entails a broad systems approach and should involve local educational institutions, ministries of education and health, local eye health organisations and international funding agencies. The process requires the development of a mutually agreed operating framework to ensure that all partners are contributing equally. Local ministries must be involved in order to ensure that the education programme and the profession are duly recognised and that the relevant systems, both structural and infrastructural, are prepared to receive the workforce.

Due attention should be given to all aspects, including the curriculum, educational resources, faculty, infrastructure and equipment. Refraction should be performed as part of a comprehensive eye health assessment and educational programmes must be formulated accordingly. Despite the lack of formal global standardisation, recent optometric developments tend to require that optometrists, as a minimum, provide ocular diagnostic services (World Council of Optometry level 3)^6^ and that they are educated within a 4- or 5-year framework. This will allow optometrists to reinforce the eye health team and other cadres to be used more effectively.

An optometry programme needs to factor in the requirements of local education and health systems, regulatory and legal frameworks, accreditation systems and quality assurance programmes. A parallel sustainable service development plan is also essential to programme success.

## Eye health education

The establishment of sustainable and comprehensive eye health services that comprise optometric care must also provide education opportunities for existing eye health workers and promote lifelong learning. For an effective eye health workforce, the key factors are the quality of the education delivered, and the trainee's commitment to professionalism and continuing education.

Education can begin at the community level with teachers, doctors, nurses and local community members participating in the detection and management of eye and vision disorders. At other levels, topical courses in relevant clinical and non-clinical areas such as interpersonal skills, management, public health, etc., need to be delivered, at scale, in an affordable and accessible way.

Current best practice approaches in education should be used to optimise teaching and learning, and effectively motivate trainees to provide quality eye care within the public health realm. For example, an eye health workforce looking to make a significant impact on rates of URE needs a broad base of competencies (e.g. in communication, leadership and advocacy) beyond the knowledge of clinical skills based on an individual patient approach.^7^

Finally, any educational programme needs to measure its impact on knowledge, attitudes and practice to ensure that the intervention is effective.^8^

## Costs and benefits

Human resource development in optometric service provision requires significant investment. The total global costs of education, including the capital costs, the cost of educating student refractive care personnel and student ophthalmic dispensers, and the cost of continuing professional development for all new personnel was estimated at US $543 million.^5^ Given the momentous loss in productivity that results from URE^5^, however, establishing optometric education systems would provide a substantial return on investment.

*Visit* **www.cehjournal.org/article/improving-access-to-refractive-and-eye-health-services/** *to read the article with references.*

